# Comprehensive Comparative Analysis of Local False Discovery Rate Control Methods

**DOI:** 10.3390/metabo11010053

**Published:** 2021-01-14

**Authors:** Shin June Kim, Youngjae Oh, Jaesik Jeong

**Affiliations:** Department of Mathematics and Statistics, Chonnam National University, Gwangju 61186, Korea; 208907@jnu.ac.kr (S.J.K.); 208816@jnu.ac.kr (Y.O.)

**Keywords:** biomarker, familywise error rate, false discovery rate, large scale inference

## Abstract

Due to the advance in technology, the type of data is getting more complicated and large-scale. To analyze such complex data, more advanced technique is required. In case of omics data from two different groups, it is interesting to find significant biomarkers between two groups while controlling error rate such as false discovery rate (FDR). Over the last few decades, a lot of methods that control local false discovery rate have been developed, ranging from one-dimensional to *k*-dimensional FDR procedure. For comparison study, we select three of them, which have unique and significant properties: Efron’s approach, Ploner’s approach, and Kim’s approach in chronological order. The first approach is one-dimensional approach while the other two are two-dimensional ones. Furthermore, we consider two more variants of Ploner’s approach. We compare the performance of those methods on both simulated and real data.

## 1. Introduction

Due to the advent of advanced high-throughput technologies, a large amount of raw data have been produced and various methods that can appropriately preprocess such data have been developed. After various preprocessing steps, statistical methods are applied to the preprocessed, yet large-scale data. When the large-scale data consists of two different groups, an interesting question is whether there are some biomarkers showing significant difference between two groups. If it is the case, then it is crucial to find significant biomarkers while controlling error rate. For example, in case of classic test for single hypothesis, it is easy not only to find biomarkers but also to control type I error, which is the probability of false rejection under the true null. However, as the number of hypotheses to be tested increases (also known as multiple testing problem), it is getting more difficult to find some biomarkers while controlling type I error at the nominal level because very small amount of error is given to test for each hypothesis depending on the number of all hypotheses. To overcome such difficulty, various approaches had been tried [1,2,3,4,5,6,7,8,9]. As a solution, the control of false discovery rate was suggested as substitute for that of type I error. Formal definition of FDR was introduced in 1995. After that, new procedures that control false discovery rate have been developed [10,11,12,13,14,15]. More specifically, in 1995, Benjamini and Hochberg (BH) firstly introduced the formal definition of FDR and proposed a step-down procedure to control FDR [10]. Especially, the procedure considered the ordered *p*-values and controlled global FDR on average sense. Following the seminal paper, FDR issue has attracted a lot of researchers’ attention. After a while, focus moved on to the local FDR combined with model-based approach. Many methodologies, which control local false discovery rate in the mixture framework,

(1) $$f\left(z\right)=\pi_{0}f_{0}\left(z\right)+\left(1-\pi_{0}\right)f_{1}\left(z\right),$$

have been developed where $f_{0}\left(z\right)$ and $f_{1}\left(z\right)$ are null and alternative densities, respectively [11,12,13,15]. $\pi_{0}$ is the proportion of true null. Furthermore, $f_{1}$ could be one-sided or two-sided. In 2001, Efron and his colleagues (called Efron hereafter) provided a new procedure that controls one-dimensional local false discovery rate,

(2) $$fdr1d\left(z\right)=\pi_{0}f_{0}\left(z\right)/f\left(z\right)$$

where *z* is test statistic [11]. In the empirical Bayes’ perspective, they interpret the fdr1d(z) as a posteriori probability of false rejection when a gene with *z* belongs to the null. After that, Ploner and his coworkers (called Ploner hereafter) firstly introduced the two-dimensional local false discovery rate to improve on the Efron’s procedure:

(3) $$fdr2d\left(z_{1},z_{2}\right)=\pi_{0}\frac{f_{0}\left(z_{1},z_{2}\right)}{f(z_{1},z_{2})},$$

where $\left(z_{1},z_{2}\right)=\left(t\;statistic,log\left(se\right)\right)$ are two-dimensional test statistic [15]. Dimension extension of test statistic caused the decrease in the number of false positives, leading to better performance. Kim and his colleagues (called Kim hereafter) also extended the dimension of test statistic, but in a different way that they apply Bonferroni correction to the combination of two marginal Efron’s local FDR [13]. Unlike Ploner, they explicitly represented two different types of composite null hypotheses: union null and intersection null (see Figure S1). Depending on the null type, they proposed different procedure that could control two-dimensional local false discovery rate.

For comparison, we here consider three different local FDR control methods: Efron, Ploner, and Kim. All methods consider mixture model as a density of test statistics. However, those methods can be differently categorized by their own properties. The Efron procedure can be used to control one-dimensional FDR while the other two (Ploner and Kim) can be applied to the control of two-dimensional FDR. Since Efron is the only method that controls one-dimensional local FDR, we here consider two more variants of Ploner. As mentioned in the literature, Ploner can be reduced into one dimensional approach through conditional expectation [15], which is called Ploner1d. Furthermore, classic two-sample *t*-statistic in Ploner1d can be replaced with Efron’s *t*, i.e., modified *t*-statistic. We call this Ploner1dE. The main difference between two 2-dimensional FDR procedures is whether the type of null matters or not. In case of Kim, the estimation of fdr2d severely depends on the type of null: union null or intersection null. However, the null type has no effect on the estimation of fdr2d in Ploner because they did not take null type into account when estimating fdr2d.

In this paper, we compare the performance of the all methods on both simulated and real data. In Section 2, we provide data analysis results which is followed by brief discussion in Section 3. We introduce the three methods in Section 4 and then provide brief conclusion in Section 5.

## 2. Results

### 2.1. Simulation Study

We consider three different scenarios: basic, mean shift, and scale change. In all scenarios, we consider the following mixture model

(4) $$f\left(z\right)=\pi_{0}f_{0}\left(z\right)+\pi_{1}f_{1}\left(z\right).$$

Throughout the simulation study, we consider $\pi_{0}=0.8$ and $f_{0}$ is the standard normal density. For comparison purpose, we generate data matrix, size of 3000 by 40, each group consisting of 20, respectively (see Figure S2). Furthermore, for each scenario, we consider two different types of true alternatives: one-sided and two-sided alternative. For one-sided alternative, right-tail alternative only is considered. In case of two-sided alternative, symmetric alternative density is considered. Even though we consider two different types of alternative, the results for two-sided alternative are more highlighted here because one-sided alternative can be considered as a subcase of two-sided alternative.

Efron is the only one-dimensional FDR control method to be considered here. However, Ploner also can be used for one-dimensional case. As mentioned in Ploner et al., $fdr1d(z_{1})$ is defined as conditional expectation of fdr2d given $Z_{1}=z_{1}$, i.e., $fdr1d\left(z_{1}\right)=E\left[fdr2d\left(z_{1},Z_{2}\right)|z_{1}\right].$ We call it Ploner1d. Furthermore, the classic *t*-statistic can be replaced with Efron’s *t*-statistic, i.e., $t^{E}$. Then we get a new estimator of local false discovery rate coupled with Efron’s *t*, which we call Ploner1dE. Therefore, three methods for the estimation of fdr1d are considered here: Efron, Ploner1d, and Ploner1dE. Furthermore, as a reference, we provide true fdr1d. The calculation of the true fdr1d is given in Section 2.4 in Supplementary file. For the estimation of fdr2d, we consider two methods: Ploner2d and Kim.

#### 2.1.1. Basic Scenario

In basic scenario, we set the problem easy to separate alternative from null. We consider two types of alternative densities, which are well-separated from the true null: one-sided or two-sided. We generate 3000×40 data matrix and then estimate FDR by using all methods. Once all estimates are given, we apply three different cutoffs (0.05,0.1,0.2) to the estimated false discovery rate. This process is repeated 100 times.

**Figure 1:**
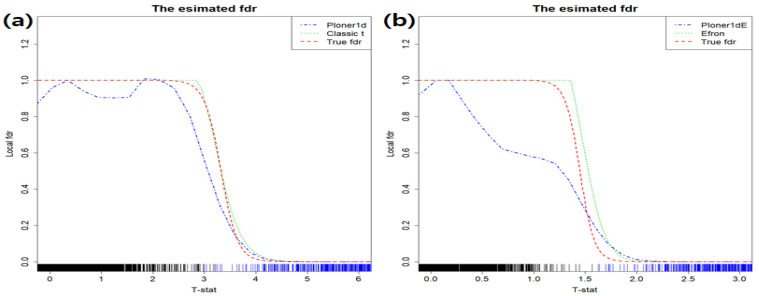
Basic scenario and one-sided alternative. Estimated fdr1ds by using classic *t*-statistic (**a**) and modified *t* (**b**). As a reference, true fdr1d is provided in red.

**One-sided alternative** In the mixture model, we set $\pi_{1}=0.2$ and about 600 rows have different group means. That is, random sample for group 1 (G1) come from $N(2.5,1.5^{2})$ and random sample for group 2 (G2) from N(0,1). The estimated fdr1ds are provided in Figure 1: the estimated fdr1d by classic *t* (left) and modified *t*-statistic (right).

The estimated fdr1d by Ploner1d is given in left panel of Figure 1 and estimated fdr1d by Efron and Ploner1dE in the right, which are represented in different colors: Efron (green), Ploner1dE (blue). As a reference, true fdr1d is presented in red. Our focus is usually on the small value of FDR, i.e., right tail of estimated fdr1d in this case. Since the true alternative density is not much overlapped with the true null density, it is easy to find biomarkers belonging to alternatives (see Figure S3). In addition, as seen in the figure above, all methods show similar performance and control nominal FDR well.

For the estimation of fdr2d, we calculated two dimensional information such as *t*-stat and log se from the same data. Depending on the null type (union or intersection null), Kim’s method provides different results. However, Ploner’s approach provides the same results regardless of the null type. In essence, the estimation in Ploner is performed on the joint distribution of two-dimensional statistics.

**Figure 2:**
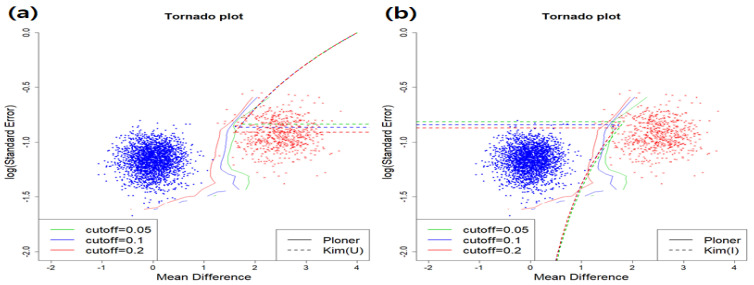
Basic scenario and one-sided alternative: estimated fdr2d for union null (**a**) and intersection null (**b**).

The estimated fdr2ds for the union null are provided in the left panel of Figure 2. Top right part of it corresponds to the rejection region by Kim while about right half of the figure is the rejection region by Ploner. True null and true alternative are represented as dots in different colors: null (blue) and alternative (red). All red dots should have been rejected here. However, by Kim, only about half of them are correctly rejected. That is, many false negatives are observed. Unlike Kim, Ploner separates true alternative (red dots) from true null (blue dots) very well. The results for the intersection null are given in Figure 2b. All areas, except for the bottom left part, correspond to the rejection region by Kim. In this case, all red dots are correctly rejected. However, only a few true nulls (blue dots) are falsely rejected by Kim, producing some false positives. To be more precise, the number of false positives for each cutoff value are 1, 17, and 26, respectively. One more thing to mention is that Ploner’s rejection region is between two of Kim’s rejection region. That is, it includes Kim’s rejection region for union null while it is included in that for intersection null.

**Two-sided alternative** In the mixture model, we set $\pi_{1}f_{1}=\pi_{11}f_{11}+\pi_{12}f_{12}$ where $\pi_{11}=\pi_{12}=0.1$. Again, about 600 rows have different group means. To be more specific, about 300 random sample for G1 come from $N(2.5,1.5^{2})$ and another 300 from $N(-2.5,1.5^{2})$, respectively. Just like the one-sided alternative, we follow the exactly same procedure with the new $\pi_{1}f_{1}$.

**Figure 3:**
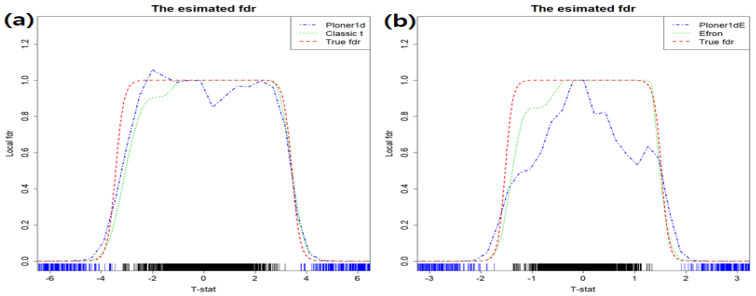
Basic scenario and two-sided. Estimated fdr1ds by using classic *t*-statistic (**a**) and modified *t* (**b**). As a reference, true fdr1d is provided in red.

In Figure 3, the estimated fdr1ds are provided in different colors. The true fdr1d, as a reference, is presented in red. The vertical lines at the bottom of the figure represent true null (black) and true alternative (blue), respectively. As seen in the figure, true alternatives are well separated from true null at any nominal FDR level.

**Figure 4:**
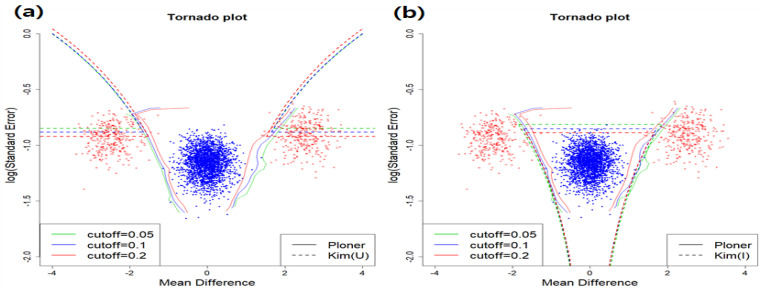
Basic scenario and two-sided. Estimated fdr2d for union null (**a**) and intersection null (**b**).

In Figure 4, we provide the estimated fdr2ds for union null (left) and intersection null (right). For union null, since we consider two-sided alternative here, two parts (top left and top right) correspond to the rejection region by Kim. Again, some red dots that should be rejected are not rejected, leading to false negatives. The number of false negatives by Kim for each cutoff value are 13, 0, and 0, respectively. However, for the intersection null, almost all true alternatives are correctly rejected.

For each method, mean and standard error of the estimated FDRs over 100 repetitions are summarized in Table 1. As seen in Table 1, all procedures control local FDR strictly.

Also, we selected one sample out of 100 and calculated some performance measures for each method when cutoff = 0.1 (Table 2). Not surprisingly, all methods in case of basic scenario show very good performance.

#### 2.1.2. Mean Shift

We now make estimation situation more difficult than the basic scenario by moving the mean of alternative density close to that of true null density, i.e., smaller margin between two group means. We consider three different mean values of alternative density, μ=(1,1.5,2). However, variance does not change here. We generate 3000×40 data matrix and then estimate local FDR by using all methods. Once all estimates are given, we apply the cutoff value of (0.05,0.1,0.2) to the estimated false discovery rate. This process is repeated 100 times. Again, we consider two types of alternative: one-sided or two-sided. As seen in the basic scenario, both one-sided and two-sided alternative show similar results and we here provide the results for two-sided alternative only. The results for one-sided alternative are provided in Supplementary file.

**Figure 5:**
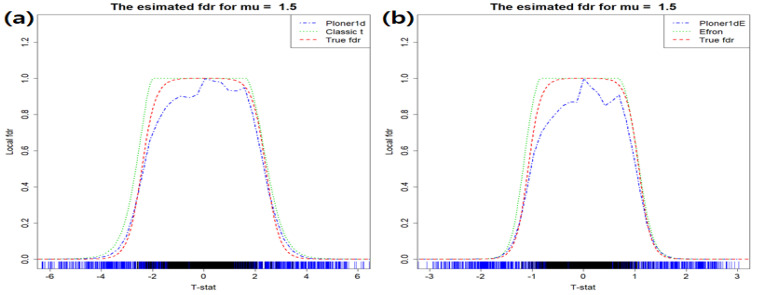
Mean shift scenario and two-sided. Estimated fdr1ds by (**a**) Classic t and Ploner1d (**b**) Efron and Ploner1dE when μ=1.5.

**Figure 6:**
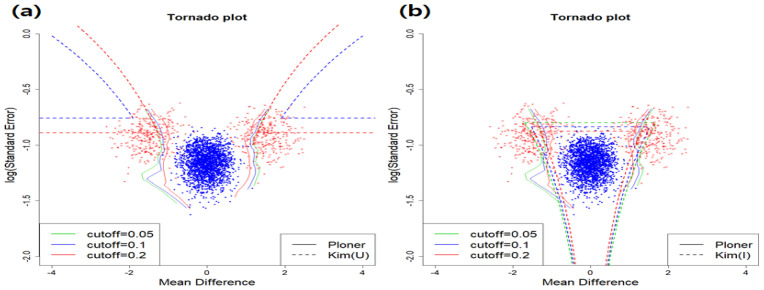
Mean shift scenario and two-sided. Estimated fdr2d when μ=1.5. (**a**) Union null (**b**) Intersection null.

**Two-sided alternative** The scheme to generate random sample is similar to the basic scenario. Only difference is that 10 percent of r.s. for G1 come from $N(\mu ,1.5^{2})$ and another 10 percent from $N(-\mu ,1.5^{2})$. Three mean values μ=(1,1.5,2) are considered. Just like basic scenario, we follow the exactly same procedure with the new $\pi_{1}f_{1}$. When μ=1.5, Figure 5 and Figure 6 include the estimated fdr1d and the estimated fdr2d, respectively.

In fdr2d estimation when μ=1.5, Kim’s intersection null (Figure 6b) rejects correctly true alternatives, i.e., red dots.

When cutoff = 0.1, means and standard errors of the estimated FDRs for each method over 100 repetitions are summarized in Table 3. As summarized in Table 3, all procedures control local FDR strictly.

**Figure 7:**
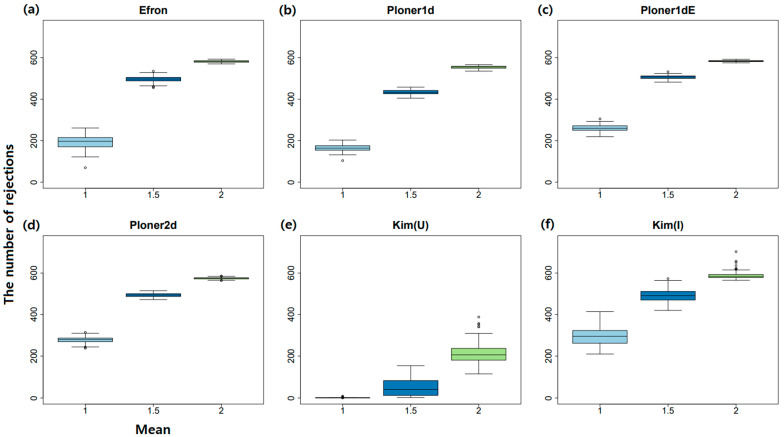
Mean shift scenario and two-sided. The number of rejections by each method over 100 repetitions when cutoff = 0.1. Box plots for three mean values by each method: (**a**) Efron (**b**) Ploner1d (**c**) Ploner1dE (**d**) Ploner2d (**e**) Kim (Union) (**f**) Kim (Intersection).

In addition, when cutoff = 0.1, box plots of the number of rejections by each method over 100 repetitions are summarized in Figure 7. As seen in the figure, the number of rejections decrease as μ decreases to keep nominal local FDR. Especially, when μ=2, i.e., case of easy separation, almost all true alternatives are correctly rejected.

In addition, we selected one sample out of 100 and calculated some performance measures for each method when μ=1.5 and cutoff = 0.1 (Table 4).

**Figure 8:**
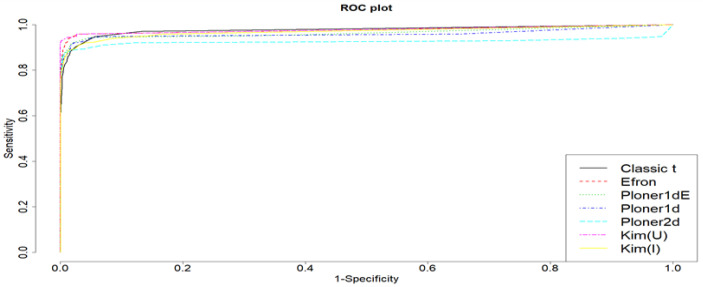
Mean shift scenario and two-sided. ROC curve when μ=1.5 and cutoff values ranging from 0 to 1 with steps of 0.05.

ROC curve when μ=1.5 is provided in Figure 8. For this, we considered various cutoff values ranging from 0 to 1 with steps of 0.05. Based on the plot, it is clear that all methods show satisfactory results.

#### 2.1.3. Scale Change

Just like the mean-shift scenario, we make estimation situation more difficult, but in a different way by increasing the variance of alternative density. More specifically, we consider three different variances of alternative density, $k\sigma^{2}$, i.e., $k=\left(2,3,4\right),\;\sigma^{2}=1.5^{2}$. We generate 3000×40 data matrix and then estimate FDR by using all methods. Once all estimates are given, we apply the cutoff value of (0.05,0.1,0.2) to the estimated false discovery rate. This process is repeated 100 times. Again, we here provide the results for two-sided alternative only. The results for one-sided alternative are provided in Supplementary file.

**Figure 9:**
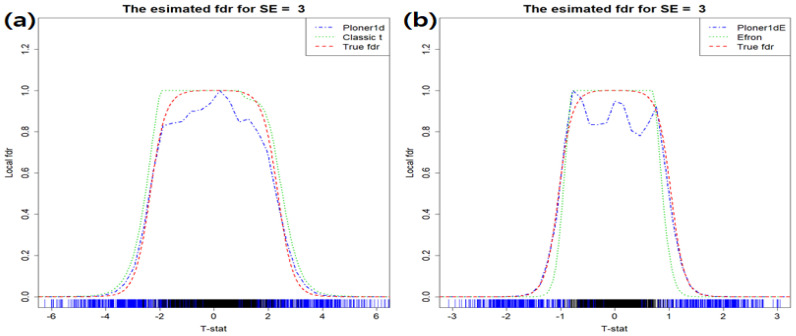
Scale change scenario and two-sided. Estimated fdr1ds by (**a**) Classic t and Ploner1d (**b**) Efron and Ploner1dE when k=4.

**Figure 10:**
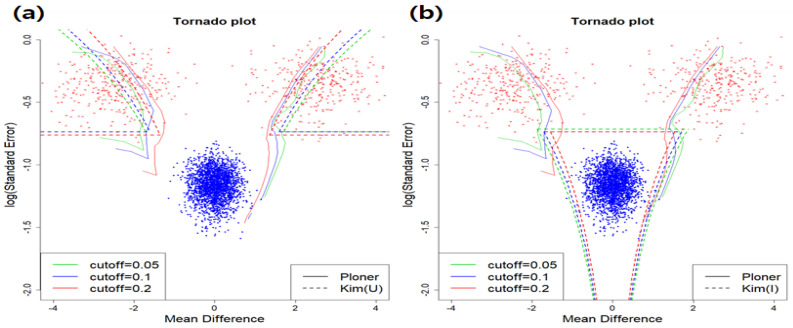
Scale change scenario and two-sided. Estimated fdr2d when k=4: (**a**) Union null (**b**) Intersection null.

**Two-sided alternative** In the mixture model, we set $\pi_{1}f_{1}=\pi_{11}f_{11}+\pi_{12}f_{12}$ where $\pi_{11}=\pi_{12}=0.1$. 10 percent of random sample come form $N(2.5,k\times 1.5^{2})$ and another 10 percent from $N(-2.5,k\times 1.5^{2})$. Three different k=(2,3,4) are considered. Just like basic scenario, we follow the exact same procedure with the new $\pi_{1}f_{1}$. When k=4, Figure 9 and Figure 10 include the estimated fdr1d and the estimated fdr2d, respectively.

When k=4, the estimated fdr2ds for union null (left) and intersection null (right) are provided in Figure 10. Different cutoff values by Kim are denoted by the dotted line in different colors: cutoff = 0.05 (green), cutoff = 0.1 (blue), and cutoff = 0.2 (red). In case of cutoff = 0.1, means and standard errors of estimated FDR over 100 repetitions are summarized in Table 5. As seen in Table 5, all procedures control local FDR strictly.

**Figure 11:**
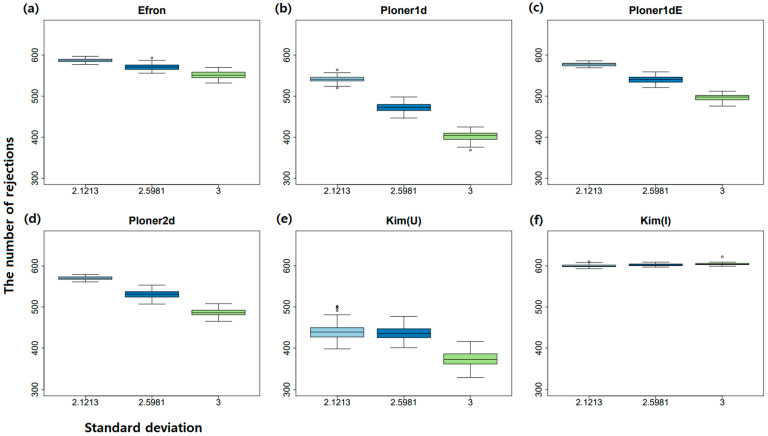
Scale change scenario and two-sided. The number of rejections by each method over 100 repetitions when cutoff = 0.1. Box plots for three mean values by each method: (**a**) Efron (**b**) Ploner1d (**c**) Ploner1dE (**d**) Ploner2d (**e**) Kim (Union) (**f**) Kim (Intersection).

In addition, when cutoff = 0.1, the box plot of number of rejections by each method over 100 repetitions are summarized in Figure 11. As seen in the figure, the number of rejections decrease as *k* increases to keep nominal local FDR. Compared to mean shift scenario, the number of rejections does not change drastically as *k* varies.

In addition, we selected one sample and calculated some performance measures for each method when k=4 and cutoff = 0.1 (Table 6). Clearly, all methods show satisfactory performance.

**Figure 12:**
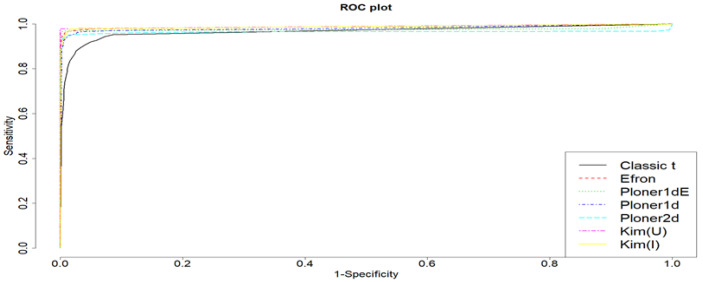
Scale change scenario and two-sided. ROC curve when k=4 and cutoff values ranging from 0 to 1 with steps of 0.05.

ROC curve when k=4 is provided in Figure 12. For this, we considered various cutoff values ranging from 0 to 1 with steps of 0.05.

### 2.2. Real Data Analysis

We investigate the performance of all methods on two different real data set: omija data [13] and lymphoma data [15]. The first data set was used by Kim et al. while the second data set was used by Ploner et al. Experimental details about two sets of real data are provided in the original literature respectively [16,17].

#### 2.2.1. Omija Data

The data matrix (3226 by 57) were obtained by using 57 *Schisandra chinesis* from two different countries: 27 species of China and 30 species of Korea. Three dimensional chromatograms were obtained by GC/MS and then converted into a data table by summing the intensities in the predetermined intervals from the instrument [13]. More details about the experiment are available in the original literature [17].

**Figure 13:**
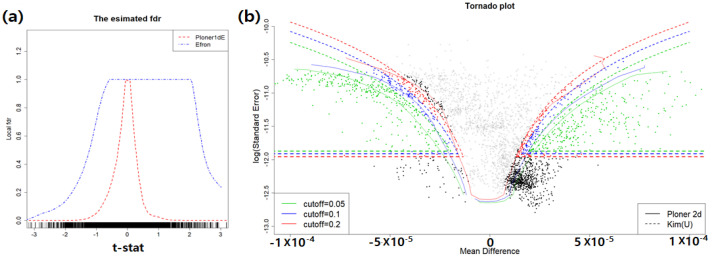
Omija data. (**a**): estimated fdr1d; (**b**): estimated fdr2d.

Two fdr1d estimates obtained by using modified *t*-statistic (left) and two fdr2d estimates (right) are provided in Figure 13.

The fdr2d estimates for union null are provided in the right panel of Figure 13. Each dot in the figure represents each retention time. Three different target FDRs are displayed in different colors: green (0.05), blue (0.1), and red (0.2). Surprisingly, we observe huge overlap at the top of two tornado plots. The dots rejected by Kim at the FDR level of 0.05 are presented in green. The Table 7 includes the number of rejection by each method.

In case of Efron, the numbers in Table 7 are the exactly same as the number of rejections given in original literature. However, for other two methods, we slightly modified the code to get the FDR estimation make more sense. Therefore, there are little difference between the numbers in Table 7 and the results in original literature.

#### 2.2.2. Lymphoma Data

Lymphochip DNA micorarrays, which consist of 12,196 clones of complementary DNA, were used to get gene expression data from 240 patients with untreated diffuse large-B-cell lymphoma [16]. In addition, outcome information of two classes is available: 102 survivors and 138 death patients. The data were available and downloaded from the website (http://llmpp.nih.gov/DLBCL).

**Figure 14:**
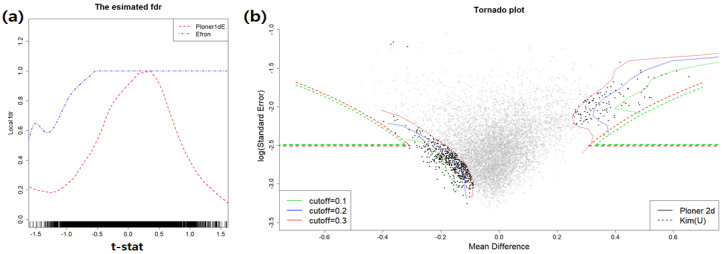
Lymphoma data. (**a**): estimated fdr1d; (**b**): estimated fdr2d.

Two different fdr1d estimates obtained by using modified *t*-statistic (left) and two different fdr2d estimates(right) are provided in Figure 14.

The fdr2d estimates for union null are provided in the right panel of Figure 14. Each gene is represented as dot in the figure. Here we consider different set of cutoff values (0.1, 0.2, 0.3). The reason is that unlike the Omija data, the number of genes rejected by all methods except for Kim(Intersection null) are almost 0. Three different cutoff values of target FDR are displayed in different colors: green (0.1), blue (0.2), and red (0.3). As seen in Table 8, there are small number of rejections by Kim (union null). The green line is exactly overlaid on the blue line and blue line is not shown in Figure 14. In contrast to the tornado plots from Omija data, there is not much overlap between two tornado plots from lymphoma data. From this, we can imagine that the results in practice heavily depend on the nature of the data. The following table includes the number of rejection.

## 3. Discussion

In this paper, we study the performance of the methods that control one-dimensional and two-dimensional local false discovery rate. It is mentioned that two-dimensional local FDR has advantages over one-dimensional local FDR [15]. More specifically, for given target FDR (say 0.1), suppose that there are two different corresponding *t*-statistics, i.e., a<b, in case of one-dimensional local false discovery rate. Then, biomarkers with *t*-statistic smaller than *a* or larger than *b* are called significant. However, in two-dimensional case, the decision on the biomarkers with the same *t*-value varies depending on the additional information, i.e., standard error. Ploner et al. also mentioned this point. For example, as seen in Figure 1d in Ploner et al., there are many genes with *t*-statistic =−3.8. Among them, the gene with log standard error =−1.0 is not rejected when the target FDR is 0.1. However, the gene with log standard error =−0.5 is rejected at the same level of FDR. Clearly, the decision on the genes with the same *t*-statistic could be different. It is also known that the small standard error inflates *t*-statistic and false positive may happen due to such small standard error. However, two-dimensional local FDR control method has a chance to figure out *t*-statistic inflated by small standard error. Hence, two-dimensional local FDR makes decision on the gene with small standard error conservatively compared to one-dimensional one. As a result, false positives could be reduced through dimension extension from 1D to 2D.

As we can imagine, the rejection region of Ploner2d is between two Kim’s rejection region. That is, we observed that the number of rejection by Ploner is smaller than Kim’s intersection null but bigger than Kim’s union null. From this, we got some insight on a new direction of future study. If we can find a new intermediate rejection region, which is the compromise of two rejection regions by Kim’s approach, new method with the intermediate rejection region would be better.

## 4. Materials and Methods

We consider methods controlling one-dimensional and two-dimensional local false discovery rate (fdr1d and fdr2d): Efron (2001) [11], Ploner (2006) [15], Kim (2018) [13]. Furthermore, there are two more variants of Ploner: Ploner1d and Ploner1dE. As mentioned earlier, Ploner1d is the method, which is reduced into one-dimensional method by conditional expectation. Ploner1dE employs the modified *t*-statistic instead of classic two-sample *t*-statistic.

### 4.1. Terminologies

Even though Soric coined the FDR terminology in 1989, Benjamini and Hochberg first defined the formal definition of the FDR ([9,10]). To introduce the FDR, the following confusion matrix is commonly used (Table 9).

It is assumed that the number of hypotheses *m* are known in advance and *R* is an observable random variable, meaning the number of hypotheses to be rejected. Then the FDR is defined as:

$$FDR=E\left(\frac{V}{R}\right),$$

while the familywise error rate (FWER) is defined:

FWER=P(V≥1).

Benjamini and Hochberg showed that the FDR is smaller than or equal to the FWER [10]. The equivalence happens only when all hypotheses are true null, $m_{0}=m$.

To define local false discovery rate, all methods to be considered here employ model-based approach. The following two-component mixture model is used and the local false discovery rate is defined as below:

$$f\left(z\right)=\pi_{0}f_{0}\left(z\right)+\left(1-\pi_{0}\right)f_{1}\left(z\right),$$

$$fdrkd\left(z\right)=\pi_{0}\frac{f_{0}\left(z\right)}{f(z)},$$

where $z={\left(z_{1},\cdots ,z_{k}\right)}^{t}$ is *k*-dimensional statistic, $f_{0}$ and $f_{1}$ are null and alternative density functions, and fdrkd denotes the estimator of *k*-dimensional local false discovery rate. In this paper, we consider k=1,2 and α is the target FDR level.

### 4.2. Efron’s Approach

Efron and his colleagues applied Bayes’s rule to the mixture model [11]. That is, they defined one-dimensional local false discovery rate as a posteriori probability of event given *z*, i.e.,

$$fdr1d\left(z\right)=P\left(NoEvent|Z=z\right)=\pi_{0}\frac{f_{0}\left(z\right)}{f(z)}=\left(\pi_{0}\left(z\right)=1-\pi_{1}\left(z\right)\right),$$

where $f\left(z\right)=\pi_{0}f_{0}\left(z\right)+\left(1-\pi_{0}\right)f_{1}\left(z\right).$ Here, *z* is the modified *t*-statistic, which is defined as

$$t_{i}^{E}=\frac{\bar{D_{i}}}{S_{i}+a_{0}},\;i=1,\cdots ,m,$$

where $\bar{D_{i}}$ is the average of paired differences, $S_{i}$ is their sample standard deviation, and $a_{0}$ is the 90th percentile of all *S* values. To find the best $a_{0}$ (90th percentile of *S* values), they searched for mappings that produce the least information loss during data reduction at the gene level, i.e., from a 320-vector in the experiment to a single number $Z_{i}.$ Such mapping also provides good separation between f(Z) and $f_{0}\left(Z\right)$. In essence, among five numbers of $a_{0}=\left(0,\;5\mathrm{th},\;50\mathrm{th},\;90\mathrm{th},\;\infty \right)$, they selected the value maximizing $\pi_{1}\left(Z\right)/\pi_{0}\left(Z\right)$, which is equivalent to maximizing $f\left(Z\right)/f_{0}\left(Z\right)$. Note that $\pi_{1}\left(Z\right)/\pi_{0}\left(Z\right)$ is the exactly same as $\left(1/\pi_{0}\right)\cdot f\left(Z\right)/f_{0}\left(Z\right)-1.$

From an estimation perspective, there are three key things: $\pi_{0},\;f_{0},$ and *f*. They first estimate the ratio $f_{0}/f$ by using logistic regression. To do so, they compute the null scores by repeatedly applying row-wise sign permutation to the difference data matrix. Since paired data was considered in the literature, they applied sign permutation. If independent sample is considered, then label permutation is typically used instead. Then the upper bound of $\pi_{0}$ is calculated:

$$\pi_{0}\leq min\left\{f\left(z\right)/f_{0}\left(z\right)\right\}.$$

Combining two estimators together, fdr1d is estimated.

Efron et al. applied the method to the paired data. Analysis results show that if fdr1d is controlled at the level of 0.1, the estimation of $\pi_{0}$ has little effect on the decision of significance. More specifically, 106 genes are called significant when using $\pi_{0}=1$ while 127 genes are called significant when using $\pi_{0}=0.811$. To find better upper bound for $\pi_{0}$, they suggest new estimate, which is defined over an interval

$$\pi_{0}\leq \frac{\int_{A}f\left(z\right)}{\int_{A}f_{0}\left(z\right)},$$

where the interval A is near z=0 (A=[−0.5,0.5] in simulation study).

Even though they applied the procedure to the paired data, transcriptional responses to ionizing radiation generated by Professor Gilbert Chu, the procedure could be generally applied to any two class problem including two sets of unpaired samples.

They also showed, from simulation study mimicking the radiation experiment, that the empirical Bayes approach is closely related to the frequentist version of FDR developed by Benjamini and Hochberg. The reason they use the terminology “empirical Bayes” is that the crucial ratio $f_{0}/f$ is estimated from the data rather than from a priori assumptions.

### 4.3. Ploner’s Approach

As seen in the Efron’s modified *t*-statistic, they tried to adjust the test statistic by adding some constant ($a_{0}$) to the denominator in order to reduce false positives. Ploner and his colleagues also noticed such phenomenon that small standard error would inflate test statistic and increase false discovery rate [15]. To remedy such problem, they extended the dimension of test statistics and local false discovery rate in two-component mixture model. The two-dimensional local false discovery rate is defined:

$$fdr2d\left(z_{1},z_{2}\right)=\pi_{0}\frac{f_{0}\left(z_{1},z_{2}\right)}{f(z_{1},z_{2})},$$

where $z_{1}$ and $z_{2}$ are *t*-statistic and log(se), respectively. Here classical *t*-statistic is used

$$t_{i}^{P}=\frac{{\bar{X}}_{i1}-{\bar{X}}_{i2}}{se_{i}},$$

with pooled standard error

$$se_{i}=\sqrt{\frac{\left(n_{1}-1\right)s_{i1}^{2}+\left(n_{2}-1\right)s_{i2}^{2}}{n_{1}+n_{2}-2}}\sqrt{1/n_{1}+1/n_{2},}$$

where ${\bar{X}}_{ij}$, $s_{ij}$ and $n_{j}$ are the gene-wise group mean, standard deviation and sample size for gene *i* and group *j* = 1, 2.

For the estimation of fdr2d, they consider a procedure requiring only a single smoothing of the ratio

$$fdr2d\left(z\right)=\pi_{0}\frac{\gamma (z)}{B(1-\gamma (z))},$$

where $\gamma \left(z\right)=Bf_{0}\left(z\right)/\left[f\left(z\right)+Bf_{0}\left(z\right)\right]$, and B(=100) is the number of permutations used to generate samples from null distribution, similar to the null score in Efron’s approach. However, group label is permuted instead of sign permutation in Efron’s paper because unpaired sample is used in this paper. Let *Z* be the m×k observation matrix from *m* genes. Each permutation of group labels generates a new dataset and statistic matrix $Z^{*}$. After *p* permutations, we have a series of $Z_{1}^{*}$, … $Z_{p}^{*}$, which represent samples of *Z* under the null. Given the observed *Z* and *p* sets of null scores $Z_{1}^{*}$, … $Z_{p}^{*}$, γ(z) is estimated by using the mixed-model approach of Pawitan (2001, Section 18.10) [18]. More specifically, similar to Efron, they pre-bin the 2D space into small interval and then perform discrete smoothing of binomial data. The smoothed estimate of $\gamma_{ij}$, which is computed by using the iteratively weighted least square algorithm, is the minimizer of the penalized log-likelihood

$$\log L\left(\gamma ,\lambda \right)=-\sum_{ij}\left\{y_{ij}\log\gamma_{ij}+\left(N_{ij}-y_{ij}\right)\log\left(1-\gamma_{ij}\right)\right\}+\lambda \sum_{\left(i,j)∼(k,l\right)}{\left(\eta_{ij}-\eta_{kl}\right)}^{2},$$

where (i,j)∼(k,l) means that (i,j) and (k,l) are primary neighbors in the 2D lattice. They assumed that the number of successes in (i,j) grid, $y_{ij}$ follows binomial distribution with success probability $\gamma_{ij}$, i.e., $y_{ij}∼Bin\left(N_{ij},\gamma_{ij}\right)$. Here $N_{ij}$ is the total number of statistic in (i,j) grid. They also addressed bias correction issue, which are caused by boundary problem and data sparsity.

For the estimation of $\pi_{0}$, they employ two different options. They use the same upper-bound estimate as used in Efron for simulation data while they use the mixture-based estimates for the real datasets, which is described in Pawitan et al. [19,20].

Theoretically, Ploner’s approach can be easily extended to the high-dimensional case (≥3). However, there exists practical difficulty of smoothing in higher dimension. Even in 2D case, they develop a procedure requiring single smoothing for γ(z). However, smoothing problem still exists. That is, what is optimal for γ(z) is not necessarily optimal for fdr2d. It is also mentioned that the fdr2d performs as well as or better than the fdr1d, in dealing with misleadingly small standard errors. For fdr1d(Z), they considered four different variants of *t*-statistic: standard *t*, Efron’s *t*, Smyth’s *t*, and SAM ([11,21,22]). Compared to the fdr1d coupled with four different *t* statistics, fdr2d has better overall performance in terms of empirical global FDR. It means that it is not necessary to modify *t*-statistic any longer. Rather, extension of the dimension of test statistic is more effective than the variation of test statistic.

### 4.4. Kim’s Approach

Ploner and his colleagues did not explicitly mention the type of null hypothesis. However, Kim and his coworkers presented two different bivariate composite null: intersection-type and union-type null (see Figure S1) [13]. For example, intersection-type composite null is represented:

$$H_{0}=H_{0,1}\cap H_{0,2},$$

where $H_{0,1}$ and $H_{0,2}$ represent each univariate marginal null. The first part $H_{0,1}:\mu_{1}=\mu_{2}$ represents equal mean and the second $H_{0,2}:\sigma^{2}=0$ represents zero variance. It is clear that the form of alternative hypothesis is affected by the type of null, accordingly the rejection region should be constructed depending on the null hypothesis.

To test for composite null, they proposed a two stage procedure. In the first stage, they adopted Efron’s procedure to control one-dimensional local false discovery rate separately in each marginal space. In the second stage, to control 2d-fdr, they combined two marginal local false discovery rates by using the concept of Bonferroni’s correction. Regardless of the type of null, the first stage is the same. However, the second stage varies depending on the type of null.

Suppose that $R_{1}$ is rejection region for $H_{0,1}$ and $R_{2}$ is rejection region for $H_{0,2}$, i.e., $P\left(R_{1}\right)=\alpha_{1}$, $P\left(R_{2}\right)=\alpha_{2}$, and $\alpha_{1}+\alpha_{2}=\alpha$. Then rejection region for intersection null is $R_{1}\cup R_{2}$. Thus, it is obvious that 2d-fdr is controlled because $P\left(R_{1}\cup R_{2}\right)\leq P\left(R_{1}\right)+P\left(R_{2}\right)=\alpha$. However, in case of union null, it is more complicated to combine two marginal 1d-fdrs into one 2d-fdr. It is clear that $P\left(R_{1}\cap R_{2}\right)\leq P\left(R_{1}\cup R_{2}\right)\leq P\left(R_{1}\right)+P\left(R_{2}\right)=\alpha$. However, in most cases, the difference between $P\left(R_{1}\right)+P\left(R_{2}\right)$ and $P(R_{1}\cap R_{2})$ is not negligible. When the difference is significant, it is obvious that decision rule would be too conservative. To avoid such problem, they tried to find optimal $\alpha_{1}$ and $\alpha_{2}$ such that

(5) $$max\left(\alpha_{1},\alpha_{2}\right)\left[1+min\left(\frac{n_{1}}{n_{12}},\frac{n_{2}}{n_{12}}\right)\right]\leq \alpha ,$$

where $n_{1},\;n_{2},\;n_{12}$ are the number of hypotheses in $R_{1},\;R_{2},\;R_{1}\cap R_{2},$ respectively. More details on the derivation of Equation (5) are provided in the original literature [13].

For the estimation of null part, $\pi_{0}$ and $f_{0}$, they applied Gaussian mixture model to the data and selected the first mixture part by using “Mclust” function in the mclust package because normality assumption was made on the null distribution [23,24]. In addition, the density *f* is estimated by kernel smoothing methods with optimal kernel. The kernel density estimator is obtained by using the “density” function in R.

Here we want to emphasize two main differences from Ploner’s method. Firstly, this procedure provides a closed-form and smooth decision rule on the bivariate test statistics, leading to easy decision for new observation. Secondly, the procedure can be applied to both type of composite null. In contrast, Ploner’s method did not clearly mention the type of null.

### 4.5. Estimation of $f_{0}\left(z\right)/f\left(z\right)$

The estimation of $f_{0}\left(z\right)/f\left(z\right)$ plays a key role in estimating local false discovery rate in the methods mentioned above. However, estimating numerator and denominator separately and then taking the ratio of them is not a good idea. Ploner and his colleagues commented on this issue: “optimal smoothing for the densities may not be optimal for the fdr because different amount of data are used.” Therefore, each method tries to estimate the ratio directly, yet in a different way.

In Efron’s approach, they calculate observed test statistic *Z* and generate *B* different sets of null statistic *z*. Each set of null statistic is generated by row-wise sign permutation (B=20 in the paper). Given *Z* and *z*, they consider *Z* and *z* as “successes” and “failures”, respectively. Then they estimate the probability

(6) $$\pi \left(z\right)=\frac{f(z)}{f\left(z\right)+Bf_{0}\left(z\right)},$$

with a natural spline with 7 degrees of freedom. In practice, they divided the range [−4, 4] into 139 equal intervals, and counted the number of values of $Z(n_{Z})$ and $z(n_{z})$ in each interval. The ratio of $n_{Z}$ to $n_{z}$ is used to estimate π(z) by logistic regression. Therefore, the estimator of $f_{0}/f$ is obtained by Equation (6)

$$\left(\frac{f_{0}}{f}\right)=\frac{1-\pi (z)}{B\pi (z)}\;\;\Rightarrow \;\;\hat{\left(\frac{f_{0}}{f}\right)}=\frac{1-\hat{\pi }\left(z\right)}{B\hat{\pi }\left(z\right)}.$$

Similarly, Ploner and his colleagues try to estimate

(7) $$\gamma \left(z\right)=1-\pi \left(z\right)=\frac{Bf_{0}\left(z\right)}{f\left(z\right)+Bf_{0}\left(z\right)},$$

and then estimate fdr2d

$$fdr2d\left(z\right)=\pi_{0}\frac{\gamma (z)}{B(1-\gamma (z))}.$$

Key difference is that Ploner et al. extended the dimension of test statistic to *k* dimension. Similar to Efron, the crucial part γ(z) is estimated by binning the data into small intervals. The discretized version of estimate $r_{ij}$ is the minimizer of the penalized log-likelihood function including single smoothing parameter.

Kim and his colleagues borrowed the idea of Efron to estimate the π(z) for marginal estimation of FDR. Furthermore, for dimension extension, they applied Bonferroni correction to the combination of two marginal FDR estimates. In addition, main difference from Ploner’s approach is that Kim et al. explicitly present two types of composite null: union and intersection null, and they propose a unified approach handling both types of null.

## 5. Conclusions

Ploner (2006) [15] commented on the issue of FDR control: “different types of *t* statistics have a little effect on the performance. However, dimension extension of statistics has a huge effect on the results”. In this paper, we assured the comments mentioned in Ploner et al. through comprehensive simulation study.

**Figure 15:**
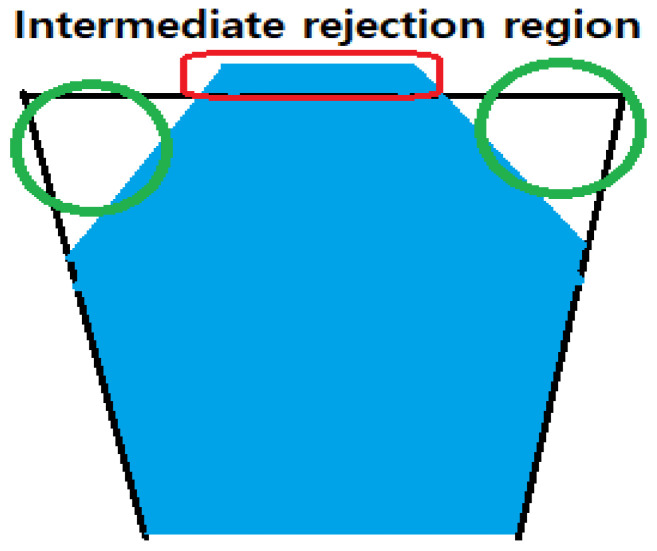
An example rejection region.

As an improvement of the Kim’s approach, we consider that combination of two rejection regions from Kim’s Union and Kim’s Intersection may produce a better rejection region. Therefore, we suggest a new rejection region (Figure 15), which improve on the control of FDR.

In case of Kim’s intersection null, some false positives were observed in the region (rectangle in red in Figure 15). In addition, false negatives were observed in the region (circle in green). Thus, we save some probability in two top corners and then get some space for the null (red rectangle area at the top). We plan to work on this issue as a direction of future study.

## Figures and Tables

**Table 1:** Basic scenario (two-sided alternative): means and standard errors of estimated FDRs over 100 repetitions.

Methods	Cutoff = 0.05	Cutoff = 0.1	Cutoff = 0.2
**Efron**	0.0003 (0.00007)	0.0004 (0.00009)	0.0011 (0.00016)
**Ploner1d**	0.0007 (0.00010)	0.0014 (0.00015)	0.0031 (0.00023)
**Ploner1dE**	0.0001 (0.00004)	0.0004 (0.00008)	0.0013 (0.00016)
**Ploner2d**	0.0005 (0.00010)	0.0017 (0.00017)	0.0051 (0.00032)
**Kim(Intersection)**	0.0047 (0.00046)	0.0133 (0.00097)	0.0342 (0.00200)

**Table 2:** Basic scenario (two-sided alternative): Performance measures when cutoff = 0.1.

	Sensitivity	Specificity	Accuracy	F1 Score
**Efron**	0.998	1	0.999	0.998
**Ploner1d**	0.997	1	0.999	0.997
**Ploner1dE**	0.982	1	0.996	0.990
**Ploner2d**	0.992	0.999	0.998	0.994
**Kim(Intersection)**	0.998	0.997	0.998	0.994

**Table 3:** Mean shift scenario (two-sided alternative): means and standard errors of estimated FDRs over 100 repetitions (cutoff = 0.1).

Methods	μ= 1	μ= 1.5	μ= 2
**Efron**	0.0114 (0.00085)	0.0086 (0.00051)	0.0026 (0.00021)
**Ploner1d**	0.0398 (0.00140)	0.0177 (0.00065)	0.0059 (0.00034)
**Ploner1dE**	0.0236 (0.00096)	0.0101 (0.00047)	0.0027 (0.00020)
**Ploner2d**	0.0110 (0.00065)	0.0052 (0.00035)	0.0028 (0.00022)
**Kim(Intersection)**	0.0316 (0.00164)	0.0220 (0.00140)	0.0202 (0.00217)

**Table 4:** Mean shift scenario (two-sided alternative): Performance measures when μ=1.5 and cutoff = 0.1.

	Sensitivity	Specificity	Accuracy	F1 Score
**Efron**	0.788	0.999	0.957	0.880
**Ploner1d**	0.800	0.999	0.959	0.887
**Ploner1dE**	0.683	0.998	0.935	0.808
**Ploner2d**	0.797	0.998	0.958	0.883
**Kim(Intersection)**	0.768	0.999	0.953	0.867

**Table 5:** Scale change scenario (two-sided alternative): means and standard errors of estimated FDRs over 100 repetitions (cutoff = 0.1).

Methods	k= 2	k= 3	k= 4
**Efron**	0.0014 (0.00018)	0.0026 (0.00025)	0.0032 (0.00027)
**Ploner1d**	0.0063 (0.00030)	0.0137 (0.00055)	0.0209 (0.00062)
**Ploner1dE**	0.0004 (0.00008)	0.0003 (0.00008)	0.0002 (0.00007)
**Ploner2d**	0.0015 (0.00017)	0.0025 (0.00023)	0.0034 (0.00030)
**Kim(Intersection)**	0.0050 (0.00033)	0.0070 (0.00036)	0.0085 (0.00044)

**Table 6:** Scale change scenario (two-sided alternative): Performance measures when k=4 and cutoff = 0.1.

	Sensitivity	Specificity	Accuracy	F1
**Efron**	0.910	0.999	0.981	0.950
**Ploner1d**	0.817	1	0.963	0.899
**Ploner1dE**	0.628	0.995	0.921	0.762
**Ploner2d**	0.793	0.997	0.956	0.879
**Kim(Intersection)**	1	0.997	0.997	0.993

**Table 7:** Omija data: The number of rejection by each method.

		1D			2D	
**Cutoff**	**Efron**	**Ploner1d**	**Ploner1dE**	**Ploner2d**	**Kim (Union)**	**Kim (Intersection)**
**0.05**	13	1425	1290	1109	636	2689
**0.1**	49	1638	1695	1355	796	2797
**0.2**	127	1906	2070	1840	963	2902

**Table 8:** Lymphoma data: The number of genes rejected by each method.

		1D			2D	
**Cutoff**	**Efron**	**Ploner1d**	**Ploner1dE**	**Ploner2d**	**Kim (Union)**	**Kim (Intersection)**
**0.1**	1	77	16	101	4	2667
**0.2**	1	298	154	370	4	3185
**0.3**	1	558	506	690	5	3569

**Table 9:** Confusion matrix when testing *m* hypotheses simultaneously.

	Decision		
	**Null**	**Alternative**	**Total**
True null	$m_{0}-V$	*V*	$m_{0}$
True alternative	*T*	R−V	$m-m_{0}$
	m−R	*R*	*m*

## Data Availability

All samples used here are available at the github website https://github.com/jjs3098/Comparison-of-FDR-control-methods.

## Supplementary Materials

The following are available online at https://www.mdpi.com/2218-1989/11/1/53/s1, Figure S1: Union null and Intersection null, Figure S2: Simulation setup: One-sided alternative and two-sided alternative, Figure S3: Basic scenario: true density, Figure S4: Mean shift scenario: True densities for μ=(1,1.5,2), Figure S5: Mean shift scenario: Estimated fdr1d for μ=1, Figure S6: Mean shift scenario: Estimated fdr1d for μ=1.5, Figure S7: Mean shift scenario: Estimated fdr1d for μ=2, Figure S8: Mean shift scenario for μ=1: Estimated fdr2d for union null and intersection null, Figure S9: Mean shift scenario for μ=1.5: Estimated fdr2d for union null and intersection null, Figure S10: Mean shift scenario for μ=2: Estimated fdr2d for union null and intersection null, Figure S11: Mean shift scenario (two-sided): True densities for μ=(1,1.5,2), Figure S12: Mean shift scenario (two-sided): Estimated fdr1d for μ=1, Figure S13: Mean shift scenario (two-sided): Estimated fdr1d for μ=1.5, Figure S14: Mean shift scenario (two-sided): Estimated fdr1d for μ=2, Figure S15: Mean shift scenario (two-sided) for μ=1: Estimated fdr2d for union and intersection null, Figure S16: Mean shift scenario (two-sided) for μ=1.5: Estimated fdr2d for union and intersection null, Figure S17: Mean shift scenario (two-sided) for μ=2: Estimated fdr2d for union and intersection null, Figure S18: Scale change scenario: True densities for k=(2,3,4), Figure S19: Scale change scenario: Estimated fdr1d for k=2, Figure S20: Scale change scenario: Estimated fdr1d for k=3, Figure S21: Scale change scenario: Estimated fdr1d for k=4, Figure S22: Scale change scenario for k=2: Estimated fdr2d for union and intersection null, Figure S23: Scale change scenario for k=3: Estimated fdr2d for union and intersection null, Figure S24: Scale change scenario for k=4: Estimated fdr2d for union and intersection null, Figure S25: Scale change scenario (two-sided): True densities for k=(2,3,4), Figure S26: Scale change scenario (two-sided): Estimated fdr1d for k=2, Figure S27: Scale change scenario (two-sided): Estimated fdr1d for k=3, Figure S28: Scale change scenario (two-sided): Estimated fdr1d for k=4, Figure S29: Scale change scenario for k=2: Estimated fdr2d for union and intersection null, Figure S30: Scale change scenario for k=3: Estimated fdr2d for union and intersection null, Figure S31: Scale change scenario for k=4: Estimated fdr2d for union and intersection null, Figure S32: Omija data: Estimated fdr2d for intersection null, Figure S33: Lymphoma data: Estimated fdr2d for intersection null, Table S1: Basic scenario (one-sided alternative): means and standard errors of estimated FDRs over 100 repetitions, Table S2: Basic scenario (one-sided alternative): Performance measure when cutoff = 0.1.

## Abbreviations

The following abbreviations are used in this manuscript:

BH	Bejamini and Hochberg
FDR	False discovery rate
FWER	Familywise error rate
2d-fdr	Two-dimensional false discovery rate
