# Supplementary material for: Comprehensive Comparative Analysis of Local False Discovery Rate Control Methods
Source: Metabolites. 2021 Jan 14;11(1):53. doi: 10.3390/metabo11010053 (PMC7830472; doi:10.3390/metabo11010053)
Supplement: Supplementary file 1 [file metabolites-11-00053-s001.pdf]

Supplementary file: Comprehensive comparative analysis of local false discovery rate control methods

Shin June Kim, Youngjae Oh and Jaesik Jeong*

1 Union and Intersection null

Kim et al. explicitly presented two different types of null: union and intersection null. Two composite nulls are graphically provided below.

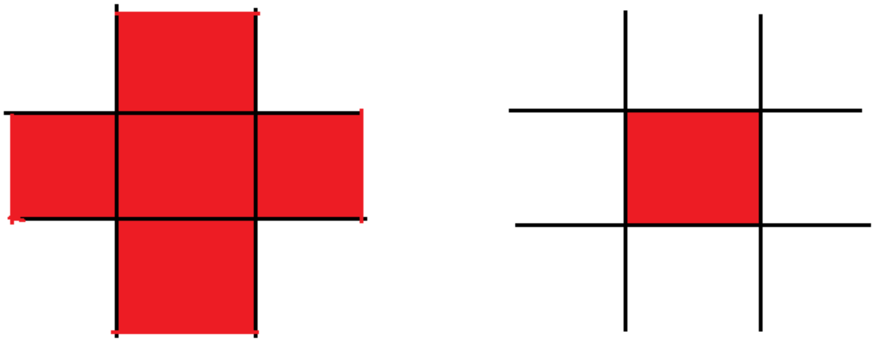

Supplementary Figure

Supplementary Figure 1: Union null (left) and Intersection null (right).

2 Simulation setup

We consider three different scenarios: basic, mean shift, and scale change. For comparison purpose, we generate data matrix, size of 3000 by 40, each group consisting of 20, respectively. Data structure for one-sided alternative is given in Fig. 2(a) and data structure for two-sided case is given in 2(b).

Throughout simulation study, we consider $\pi_0 = 0.8$ and the standard normal density is used as null density. Furthermore, for each scenario, we consider two different true alternatives: one-sided and two-sided alternative. In case of one-sided alternative, 20 percent of one group belongs to alternative, which is highlighted in red in Fig. 2(a). That is, they are generated from $N(\mu, \sigma^2)$ where $\mu > 0$. In addition, for two-sided alternative, 10 percent of

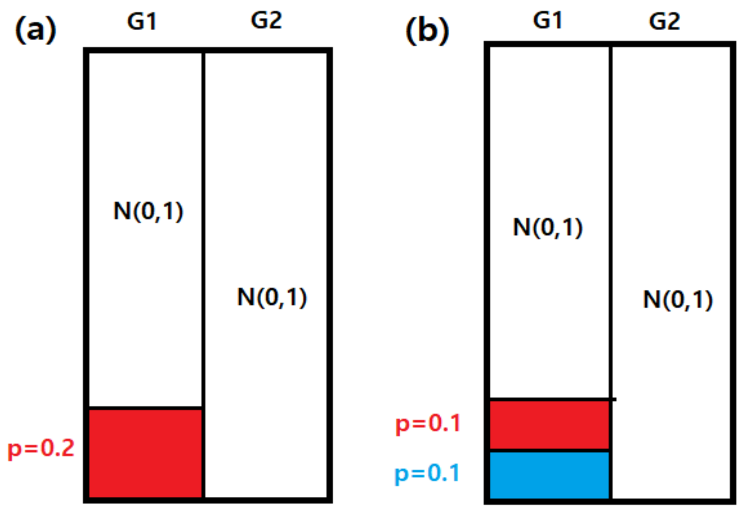

Supplementary Figure 2: Simulation setup: One-sided alternative (left) and two-sided alternative (right).

one group is generated from $N(\mu, \sigma^2)$ where $\mu > 0$ and the other 10 percent from $N(-\mu, \sigma^2)$. In all scenarios, the size of data is 3000×40 and this process is repeated 100 times.

2.1 Basic scenario

To generate sample for alternative density, $\mu = 2.5$ and $\sigma^2 = 1.5^2$ are used. We generate 3000×40 data matrix and then estimate FDR by using all methods. Three different cutoffs $(0.05, 0.1, 0.2)$ are applied to the estimated false discovery rate.

2.2 Mean shift scenario

To generate sample for alternative density, three different means, $\mu = (1, 1.5, 2)$ are considered. However, variance does not change here. We generate 3000×40 data matrix and then estimate local FDR by using all methods. Three cutoff values of $(0.05, 0.1, 0.2)$ are applied to the estimated false discovery rate.

2.3 Scale change scenario

Unlike the mean-shift scenario, we change the variance of alternative density. To generate sample for alternative density, three different variances of alternative density, $k\sigma^2$, i.e., $k = (2, 3, 4)$, $\sigma^2 = 1.5^2$. We generate 3000×40 data matrix and then estimate FDR by using all methods. Three cutoff values of $(0.05, 0.1, 0.2)$ are applied to the estimated false discovery rate.

2.4 Calculation of true fdr

We explain here how to calculate true fdr for one-sided alternative only. For simplicity, we consider basic scenario only. Among 3000 rows, there is no difference between two groups for 2400 rows. For example, let $X_1, \cdots, X_{20}$ be random sample from $N(0,1)$ and $Y_1, \cdots, Y_{20}$ be random sample from $N(0,1)$. Then both $\bar{X}$ and $\bar{Y}$ follow $N(0, 1/20)$ independently. Thus,

$$Z = \frac{\bar{X} - \bar{Y}}{\sqrt{1/10}} \sim N(0,1)$$

For other 600 rows, let $X_1, \cdots, X_{20}$ be random sample from $N(2.5, 1.5^2)$ and $Y_1, \cdots, Y_{20}$ be random sample from $N(0,1)$. Then $\bar{Y}$ follows $N(0, 1/20)$ and $\bar{X}$ follows $N(\mu, 1.5^2/20)$. By distribution theory, $\bar{X} - \bar{Y} \sim N(\mu, (1.5^2 + 1)/20)$. Thus,

$$Z = \frac{\bar{X} - \bar{Y}}{\sqrt{(1.5^2+1)/20}} \sim N\left(\frac{\mu}{\sqrt{(1.5^2+1)/20}}, 1\right)$$

As a distribution of all Z values, we can consider the following normal mixture model

$$f(z) = 0.8 f_0(z) + 0.2 f_1(z)$$

where f_0 and f_1 are density of $N(0,1)$ and $N\left(\frac{\mu}{\sqrt{(1.5^2+1)/20}}, 1\right)$, respectively. Therefore, true fdr is

$$fdr(z) = 0.8 \frac{f_0(z)}{f(z)}.$$

3 Results

3.1 Simulation results

For the estimation of fdr1d, three methods are considered: Efron, Ploner1d, and Ploner1dE. For the estimation of fdr2d, we consider two methods such as Ploner2d, and Kim.

3.1.1 Basic scenario

We consider two types of alternative densities, which are well-separated from the true null: one-sided or two-sided. For one-sided alternative, right-tail alternative only is considered. In case of two-sided alternative, symmetric alternative density is considered. We generate 3000×40 data matrix and then estimate FDR by using all methods. Three different cutoffs $(0.05, 0.1, 0.2)$ are applied to the estimated false discovery rate.

One-sided alternative We set $\pi_1 = 0.2$. To generate sample, $N(0,1)$ and $N(2.5, 1.5^2)$ are used as null and alternative density, respectively. The true density, which generate random samples, is given in Fig. 3

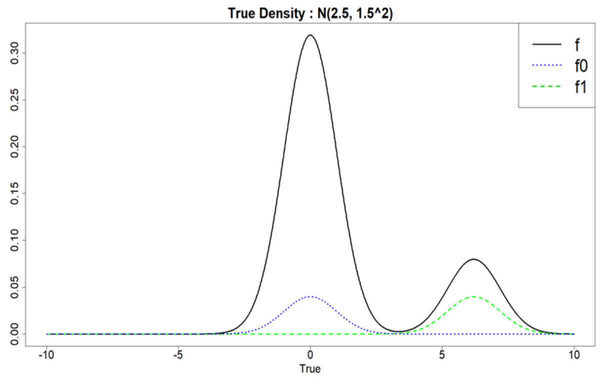

Supplementary Figure 3: Basic scenario: true density.

Figure 1 in the main text includes estimated fdr1d. Furthermore, Figure 2 in the main text includes estimated fdr2d: union null(left) and intersection null(right).

For each method, mean and standard error of the estimated FDRs over 100 repetitions are summarized in Table 1. As seen in Table 1, all procedures control local FDR strictly.

Supplementary Table 1: Basic scenario (one-sided alternative): means and standard errors of estimated FDRs over 100 repetitions.

**Cutoff**	**0.05**	**0.1**	**0.2**
**Efron**	0.0002(0.00006)	0.0004(0.00009)	0.0009(0.00015)
**Ploner1d**	0.0006(0.00009)	0.0010(0.00013)	0.0022(0.00020)
**Ploner1dE**	0.0001(0.00003)	0.0003(0.00007)	0.0020(0.00026)
**Ploner2d**	0.0014(0.00014)	0.0025(0.00021)	0.0077(0.00042)
**Kim(Intersection)**	0.0059(0.00068)	0.0155(0.00154)	0.0400(0.00356)

Also, we selected one sample out of 100 and calculated some performance measures for each method when cutoff=0.1 (Table 2). Not surprisingly, all methods in case of basic scenario show very good performance.

Supplementary Table 2: Basic scenario (one-sided alternative): Performance measures when cutoff=0.1.

	**Sensitivity**	**Specificity**	**Accuracy**	**F1 score**
**Efron**	0.997	0.999	0.999	0.997
**Ploner1d**	0.993	1	0.998	0.996
**Ploner1dE**	0.985	0.998	0.996	0.989
**Ploner2d**	0.987	0.999	0.997	0.992
**Kim(Intersection)**	0.995	0.998	0.998	0.994

Two-sided alternative To generate alternative sample, $N(2.5, 1.5^2)$ and $N(-2.5, 1.5^2)$ are used, i.e., 10 percent from each density. Figure 3 in the main text includes the estimated

fdr1d and Figure 4 in main text includes fdr2d for union null (left) and intersection null (right).

3.1.2 Mean shift scenario

As mean value of alternative density, three different means, $\mu = (1, 1.5, 2)$ are considered. However, variance does not change here. Again, we consider two types of alternative: one-sided or two-sided. We generate 3000×40 data matrix and then estimate local FDR by using all methods. Three cutoff values of $(0.05, 0.1, 0.2)$ are applied to the estimated false discovery rate.

One-sided alternative In the mixture model, we set $\pi_1 = 0.2$ and random sample for G1 come from $N(\mu, 1.5^2)$. Three different $\mu = (1, 1.5, 2)$ are considered. Fig. 4 includes the true density, which are used to generate random samples for each mean value.

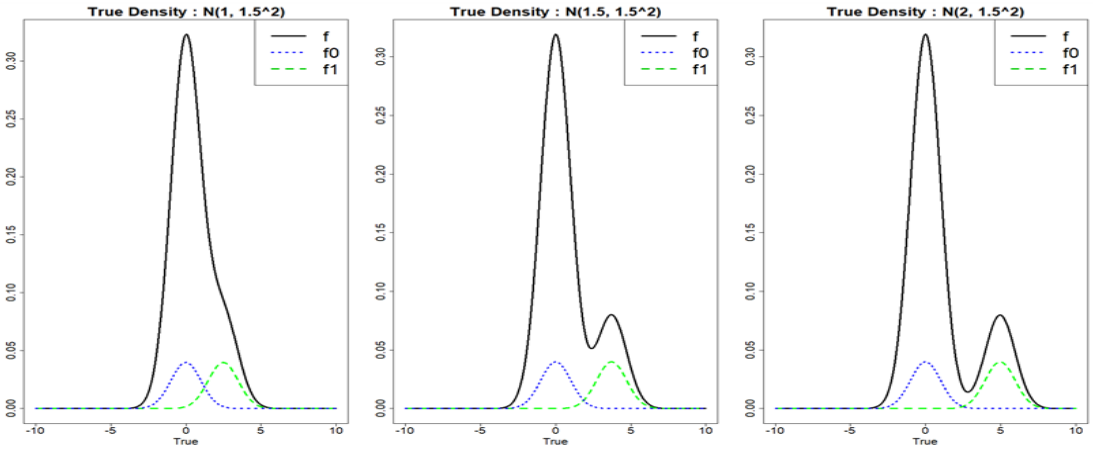

Supplementary Figure 4: Mean shift scenario: True densities for $\mu_1 = (1, 1.5, 2)$.

Fig. 5 includes estimated fdr1d when $\mu = 1$. Figures 6 and 7 correspond to $\mu = 1.5$ and $\mu = 2$, respectively. Compared to the results from Basic scenario, we observe relatively small margin between true fdr and estimated fdr1d in the mean shift scenario.

Three estimated fdr1d for $\mu = 1$ are provided in Fig. 5

Three estimated fdr1d for $\mu = 1.5$ are provided in Fig. 6

Three estimated fdr1d for $\mu = 2$ are provided in Fig. 7

When $\mu_1 = 1$, the estimated fdr2d are provided in Fig. 8

When $\mu_1 = 1.5$, the estimated fdr2d are provided in Fig. 9

When $\mu_1 = 2$, the estimated fdr2d are provided in Fig. 10

Two-sided alternative To generate alternative sample, $N(\mu, 1.5^2)$ and $N(-\mu, 1.5^2)$ are used, i.e., 10 percent from each density. Three mean values $\mu_1 = (1, 1.5, 2)$ are considered. Figures 5 and 6 in the main text includes the estimated fdr1d and fdr2d: union null (left) and intersection null (right). Three true densities are provided in Fig. 11

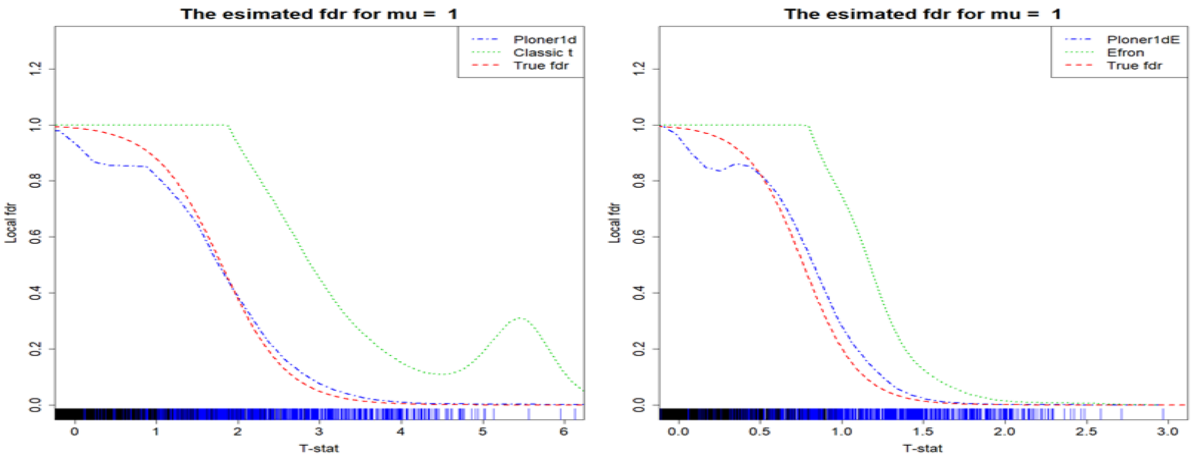

Supplementary Figure 5: Mean shift scenario: Estimated fdr1d for $\mu = 1$.

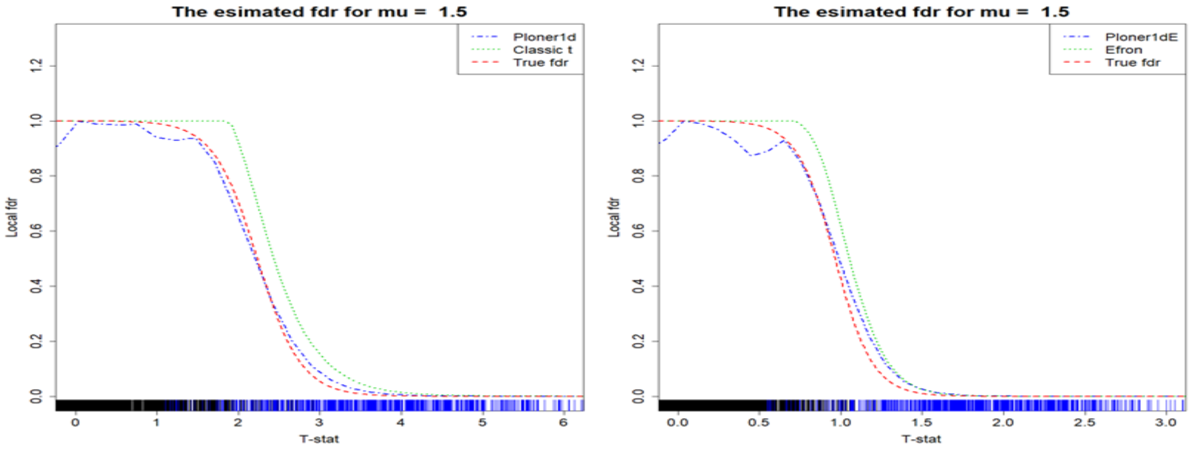

Supplementary Figure 6: Mean shift scenario: Estimated fdr1d for $\mu = 1.5$.

Three estimated fdr1d when $\mu = 1$ are provided in Fig. 12
Three estimated fdr1d when $\mu = 1.5$ are provided in Fig. 13
Three estimated fdr1d when $\mu = 2$ are provided in Fig. 14
When $\mu_1 = 1$, the estimated fdr2d are provided in Fig. 15
When $\mu_1 = 1.5$, the estimated fdr2d are provided in Fig. 16
When $\mu_1 = 2$, the estimated fdr2d are provided in Fig. 17

3.1.3 Scale change scenario

Unlike the mean-shift scenario, we change the variance of alternative density. More specifically, we consider three different variances of alternative density, $k\sigma^2$, i.e., $k = (2, 3, 4)$, $\sigma^2 = 1.5^2$. Again, we consider two types of alternative: one-sided or two-sided. We generate

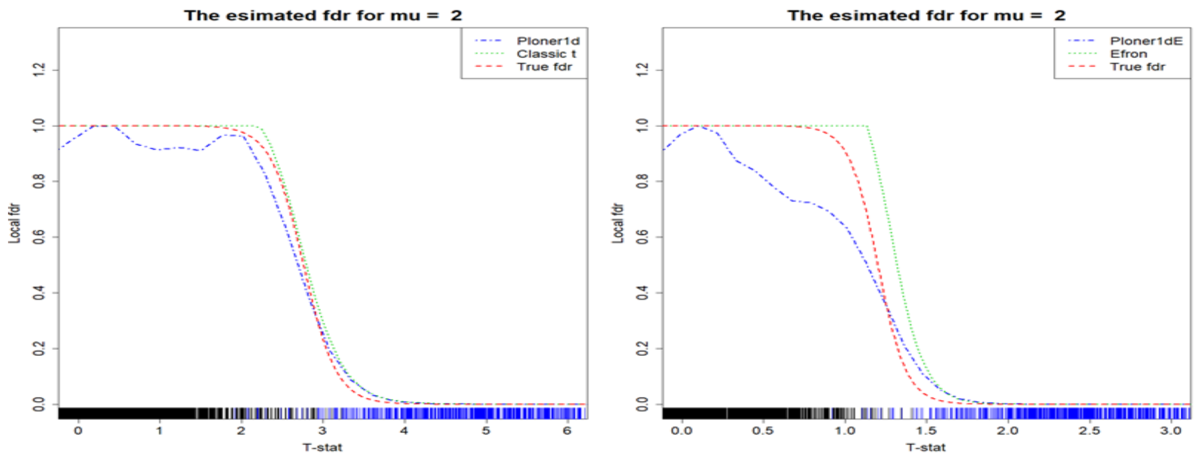

Supplementary Figure 7: Mean shift scenario: Estimated fdr1d for $\mu = 2$.

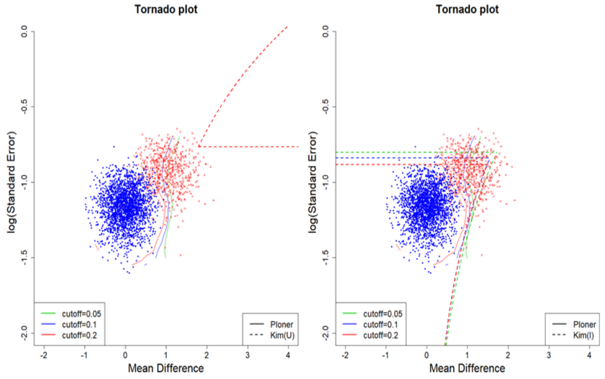

Supplementary Figure 8: Mean shift scenario for $\mu_1 = 1$: Estimated fdr2d for union null (left) and intersection null (right).

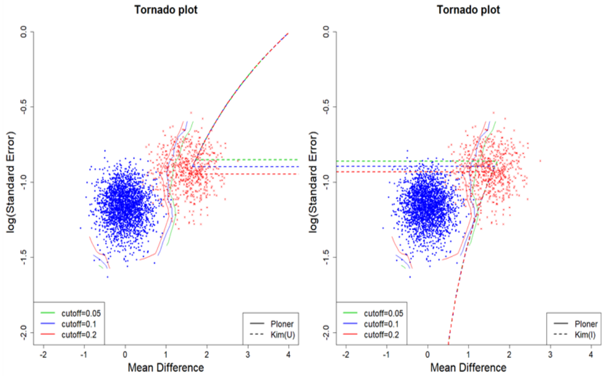

Supplementary Figure 9: Mean shift scenario for $\mu_1 = 1.5$: Estimated fdr2d for union null (left) and intersection null (right).

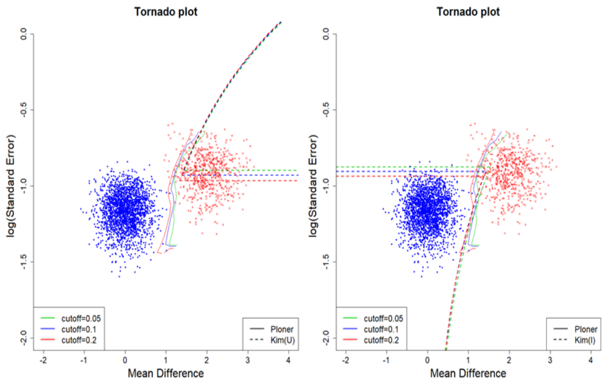

Supplementary Figure 10: Mean shift scenario for $\mu_1 = 2$: Estimated fdr2d for union null (left) and intersection null (right).

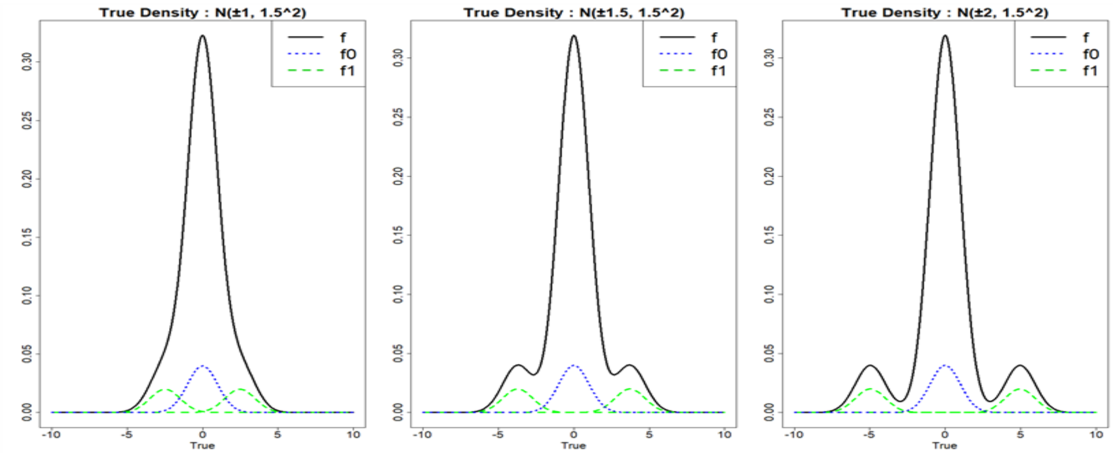

Supplementary Figure 11: Mean shift scenario(two-sided): True densities for $\mu_1 = (1, 1.5, 2)$.

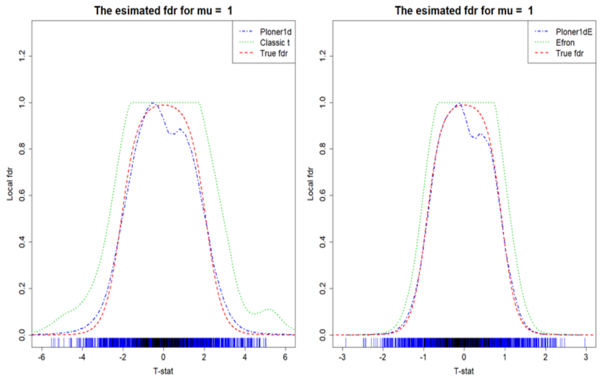

Supplementary Figure 12: Mean shift scenario(two-sided): Estimated fdr1d for $\mu = 1$.

3000×40 data matrix and then estimate FDR by using all methods. Three cutoff values of

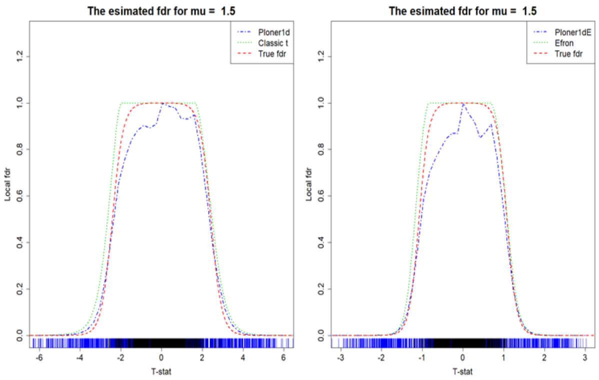

Supplementary Figure 13: Mean shift scenario(two-sided): Estimated fdr1d for $\mu = 1.5$.

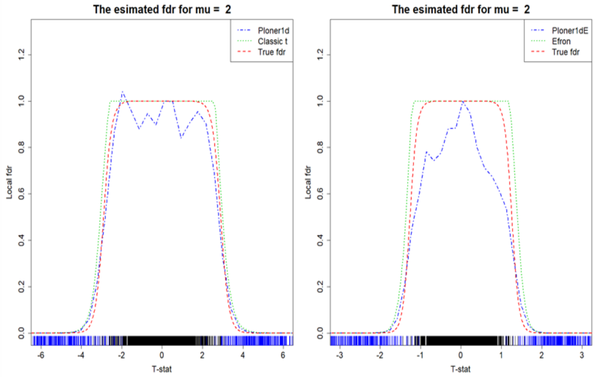

Supplementary Figure 14: Mean shift scenario(two-sided): Estimated fdr1d for $\mu = 2$.

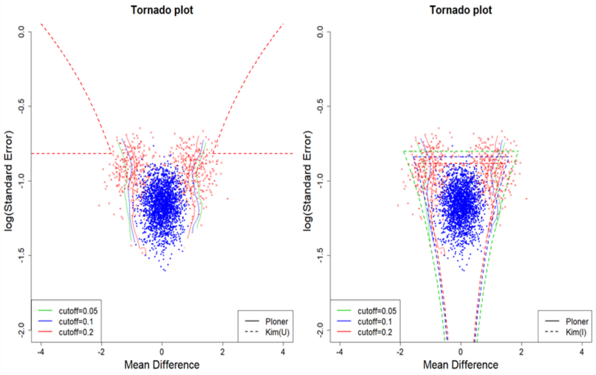

Supplementary Figure 15: Mean shift scenario(two-sided) for $\mu_1 = 1$: Estimated fdr2d for union null (left) and intersection null (right).

$(0.05, 0.1, 0.2)$ are applied to the estimated false discovery rate.

One-sided alternative In the mixture model, we set $\pi_1 = 0.2$ and random sample for G1

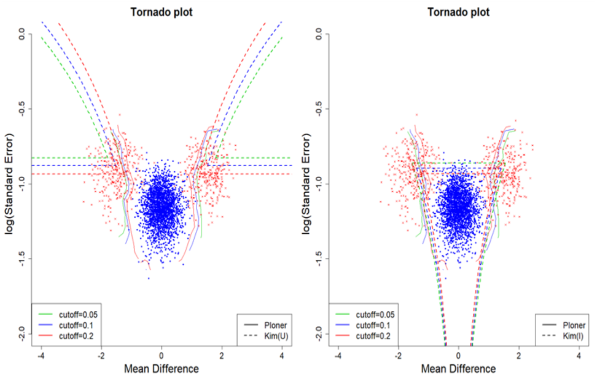

Supplementary Figure 16: Mean shift scenario(two-sided) for $\mu_1 = 1.5$: Estimated fdr2d for union null (left) and intersection null (right).

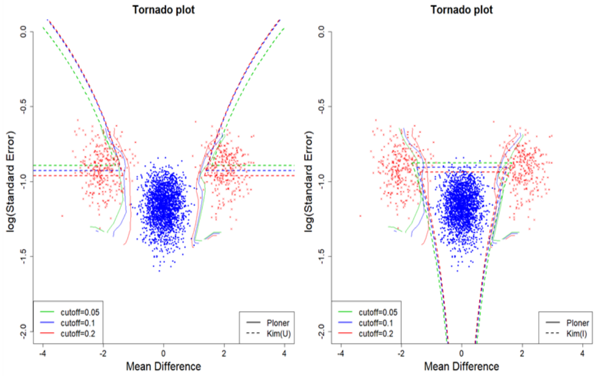

Supplementary Figure 17: Mean shift scenario(two-sided) for $\mu_1 = 2$: Estimated fdr2d for union null (left) and intersection null (right).

come from $N(2.5, k \times 1.5^2)$. Three different $k = (2, 3, 4)$ are considered. Compared to Basic scenario, there is relatively small margin between true fdr and estimated fdr1d. Three true densities are provided in Fig. 18

The estimated fdr1d when $k = 2$ are provided in Fig. 19

The estimated fdr1d when $k = 3$ are provided in Fig. 20

The estimated fdr1d when $k = 4$ are provided in Fig. 21

When $k = 2$, the estimated fdr2d are provided in Fig. 22

When $k = 3$, the estimated fdr2d are provided in Fig. 23

When $k = 4$, the estimated fdr2d are provided in Fig. 24

Two-sided alternative To generate alternative sample, $N(2.5, k \times 1.5^2)$ and $N(-2.5, k \times 1.5^2)$ are used. Three different $k = (2, 3, 4)$ are considered. Figures 9 and 10 in the main text include the estimated fdr1d and fdr2d, respectively. Three true densities are provided in Fig. 25

Three estimated fdr1d when $k = 2$ are provided in Fig. 26

Three estimated fdr1d when $k = 3$ are provided in Fig. 27

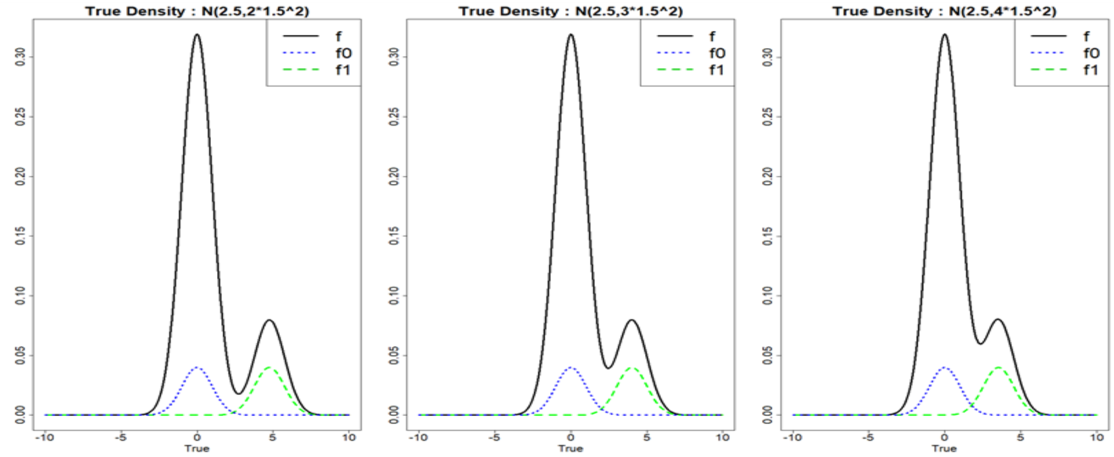

Supplementary Figure 18: Scale change scenario: True densities for $k = (2, 3, 4)$.

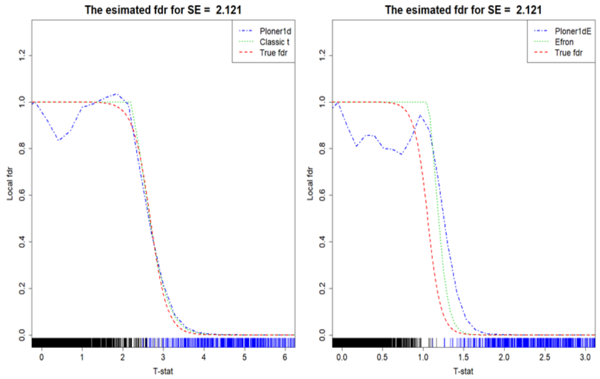

Supplementary Figure 19: Scale change scenario: Estimated fdr1d for $k = 2$.

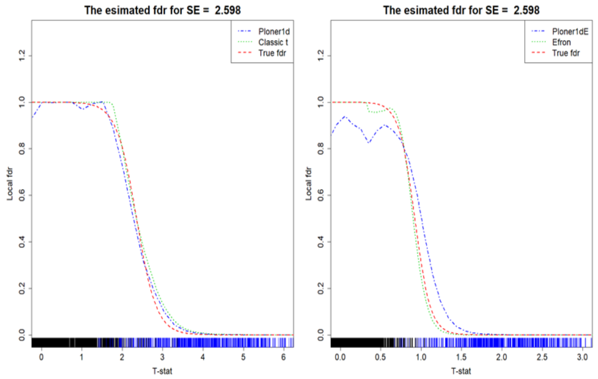

Supplementary Figure 20: Scale change scenario: Estimated fdr1d for $k = 3$.

Three estimated fdr1d when $k = 4$ are provided in Fig. 28
When $k = 2$, the estimated fdr2d are provided in Fig. 29

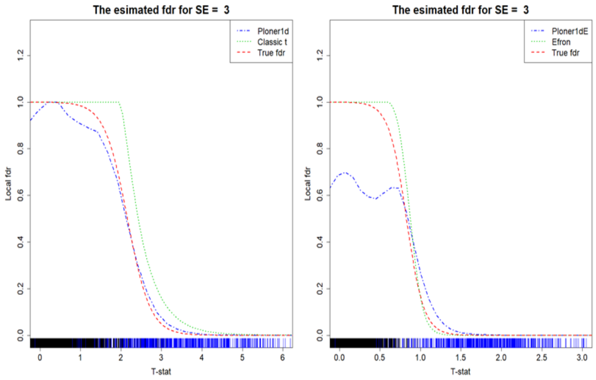

Supplementary Figure 21: Scale change scenario: Estimated fdr1d for $k = 4$.

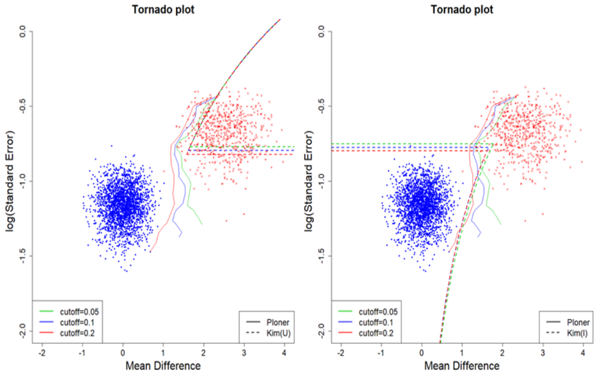

Supplementary Figure 22: Scale change scenario for $k = 2$: Estimated fdr2d for union null (left) and intersection null (right).

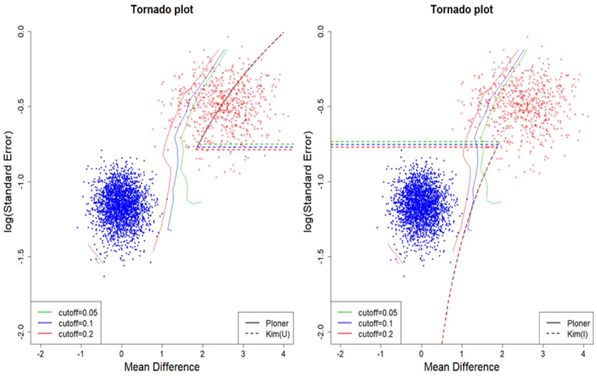

Supplementary Figure 23: Scale change scenario for $k = 3$: Estimated fdr2d for union null (left) and intersection null (right).

When $k = 3$, the estimated fdr2d are provided in Fig. 30
When $k = 4$, the estimated fdr2d are provided in Fig. 31

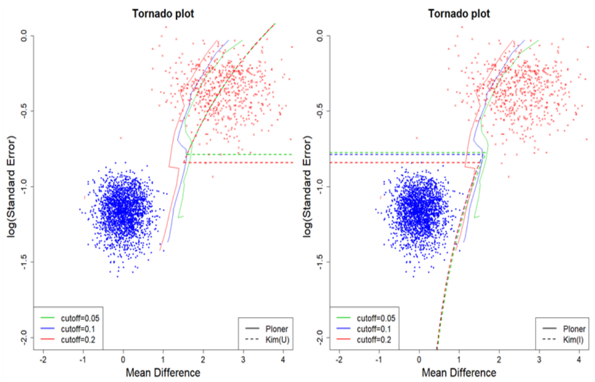

Supplementary Figure 24: Scale change scenario for $k = 4$: Estimated fdr2d for union null (left) and intersection null (right).

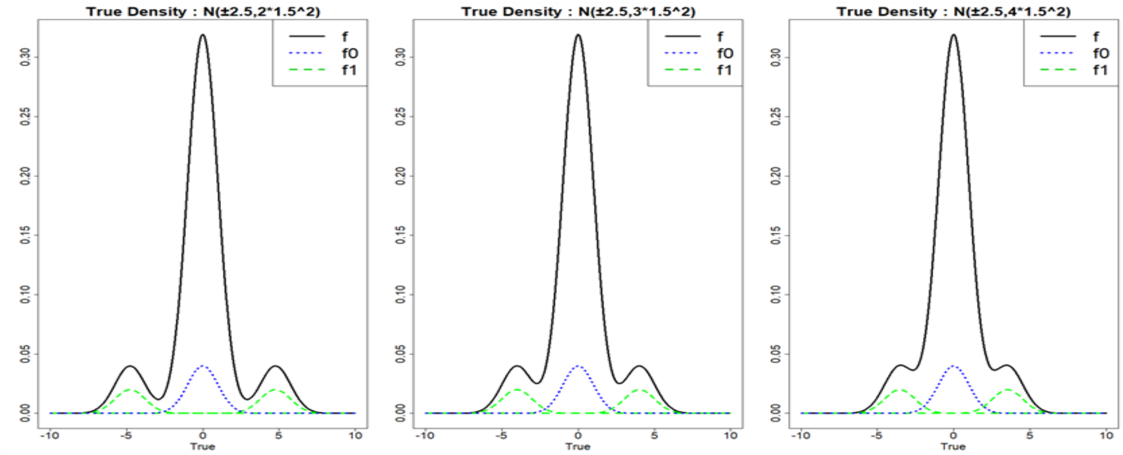

Supplementary Figure 25: Scale change scenario(two-sided): True densities for $k = (2, 3, 4)$.

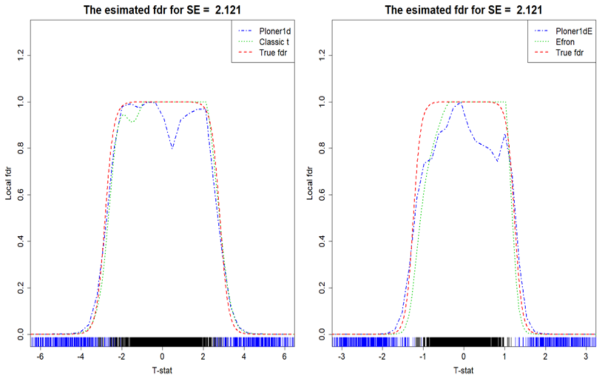

Supplementary Figure 26: Scale change scenario(two-sided): Estimated fdr1d for $k = 2$.

3.2 Real data

We investigate the performance of all methods on two different real data set: omija data and lymphoma data. The first data set was used by Kim et al. while the second data set was

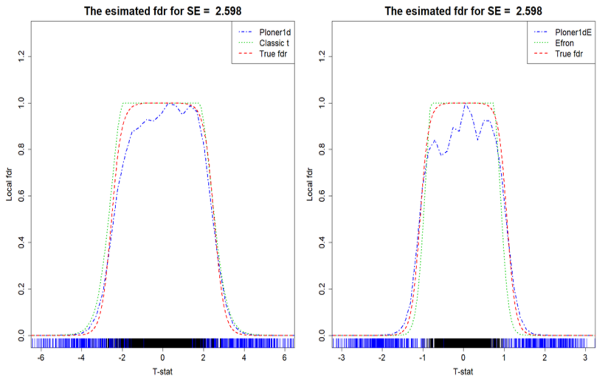

Supplementary Figure 27: Scale change scenario(two-sided): Estimated fdr1d for $k = 3$.

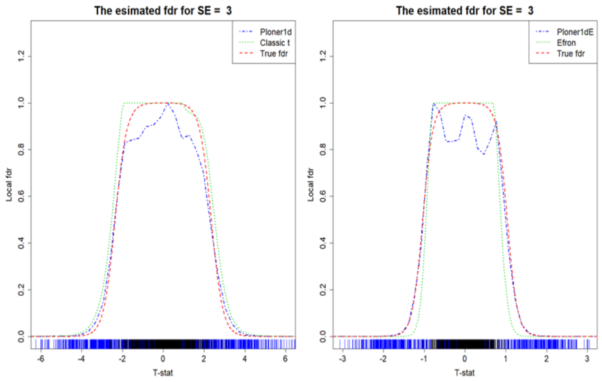

Supplementary Figure 28: Scale change scenario(two-sided): Estimated fdr1d for $k = 4$.

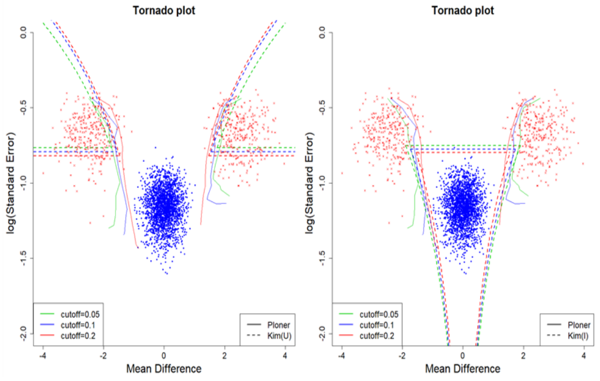

Supplementary Figure 29: Scale change scenario for $k = 2$: Estimated fdr2d for union null (left) and intersection null (right).

used by Ploner et al.

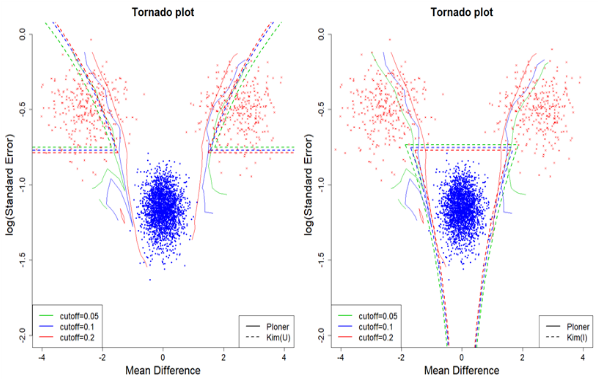

Supplementary Figure 30: Scale change scenario for $k = 3$: Estimated fdr2d for union null (left) and intersection null (right).

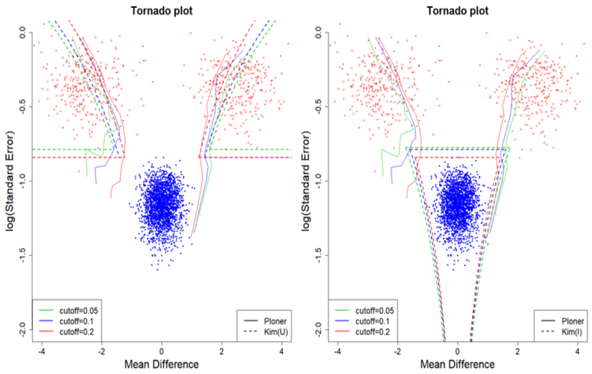

Supplementary Figure 31: Scale change scenario for $k = 4$: Estimated fdr2d for union null (left) and intersection null (right).

3.2.1 Omija data

The data matrix (3226 by 57) were obtained by using 57 schisandra chinesis from two different countries: 27 species of China and 30 species of Korea. The estimated fdr1d and estimated fdr2d for union null are provided in Figure 13 in the main text. Here we provide estimated fdr2d for intersection null in Fig. 32

3.2.2 Lymphoma data

Lymphochip DNA micorarrays, which consist of 12196 clones of complementary DNA, were used to get gene expression data from 240 patients with untreated diffuse large-B-cell lymphoma. Also, outcome information of two classes is available: 102 survivors and 138 death patients. The estimated fdr1d and estimated fdr2d for union null are provided in Figure 14 in the main text. Here we provide estimated fdr2d for intersection null in Fig. 33

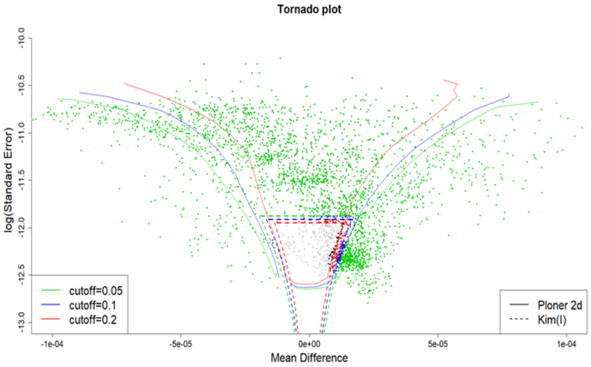

Supplementary Figure 32: Omija data: Estimated fdr2d for intersection null.

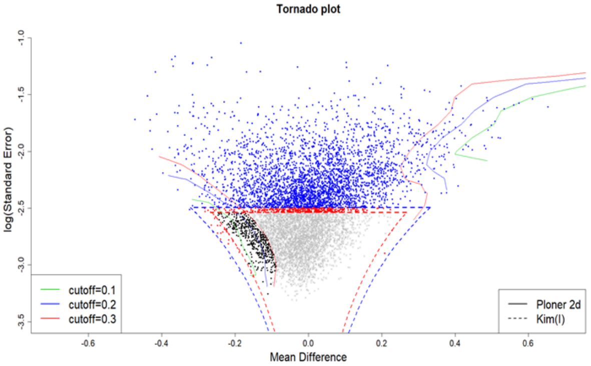

Supplementary Figure 33: Lymphoma data: Estimated fdr2d for intersection null.
